# Retraction Note: Guangxi cobra venom-derived NGF promotes the osteogenic and therapeutic effects of porous BCP ceramic

**DOI:** 10.1038/s12276-023-00931-3

**Published:** 2023-01-20

**Authors:** Pan Jin, Fuqiang Yin, Li Huang, Li Zheng, Jinmin Zhao, Xingdong Zhang

**Affiliations:** 1 Guangxi Engineering Center in Biomedical Materials for Tissue and Organ Regeneration, The First Affiliated Hospital of Guangxi Medical University, Nanning, People’s Republic of China; 2 Guangxi Collaborative Innovation Center for Biomedicine, The First Affiliated Hospital of Guangxi Medical University, Nanning, People’s Republic of China; 3 Guangxi Key Laboratory of Regenerative Medicine, The First Affiliated Hospital of Guangxi Medical University, Nanning, People’s Republic of China; 4 The Medical and Scientific Research Center, Guangxi Medical University, Nanning, People’s Republic of China; 5 National Engineering Research Center for Biomaterials, Sichuan University, Chengdu, People’s Republic of China

Retraction to: *Experimental and Molecular Medicine* 10.1038/emm.2016.173, published online 07 April 2017

The Editor in Chief has retracted the article. After publication, concerns were raised about unexpected overlaps in Fig. 2. These were addressed by two corrections. However, further concerns were identified specifically, dealing with unexpected overlaps in Figs 2CE, 3D, 4A, 6BC. The authors were unable to provide a sufficient explanation. Therefore, the Editor in Chief has lost confidence in the integrity of the article’s findings. Pan Jin and Li Zheng agree to this retraction. Fuqiang Yin, Li Huang, Jinmin Zhao and Xingdong Zhang did not respond to any correspondence from the Editor about this retraction.

